# Contrast-Enhanced Ultrasound Findings of Hepatocellular Carcinoma With Neuroendocrine Carcinoma: A Case Report

**DOI:** 10.3389/fmed.2021.602346

**Published:** 2021-08-16

**Authors:** Hong Wang, Dan Yang, Zhenru Wu, Yan Luo, Wenwu Ling

**Affiliations:** ^1^Department of Ultrasound, West China Hospital of Sichuan University, Chengdu, China; ^2^Key Laboratory of Transplantation Engineering and Transplantation Immune, The Ministry of Health, West China Hospital of Sichuan University, Chengdu, China; ^3^Laboratory of Pathology, West China Hospital of Sichuan University, Chengdu, China

**Keywords:** contrast-enhanced ultrasonography, liver, neuroendocrinal tumors, HCC, DIAG (diagnostic imaging)

## Abstract

Hepatocellular carcinoma (HCC) with the concurrent occurrence of primary hepatic neuroendocrine carcinoma (NEC) of the liver is extremely rare. Preoperative diagnosis of HCC combined with NEC is very difficult. Here, we presented a case report of HCC combined with NEC. A 33-year-old male was admitted to our hospital due to focal liver lesion. To further the diagnosis, he received laboratory tests, conventional ultrasound, contrast-enhanced ultrasound (CEUS), and magnetic resonance imaging (MRI). Grayscale ultrasound showed a hypoechoic nodule with peripheral hypoechoic halo and central small patches of anechoic area in the hepatic segment VI, and the liver background was cirrhosis. In the CEUS, the solid component of the nodule was rapidly homogeneous hyper-enhancement in the arterial phase. Then, the enhancement of the nodule was washed out slowly and gradually; the nodule presented heterogeneously mild enhancement in the portal phase, and slight hypo-enhancement was showed in the late phase. The hypo-enhanced area was mainly located in the surrounding space. Meanwhile, there was a small piece of non-enhanced area within the lesion center in the whole three-phase imaging. MRI showed a lobular contoured mass in segment VI. The patient underwent middle liver resection, splenectomy, and cholecystectomy. The pathological diagnosis was a HCC with NEC. At the time of the preparation of this manuscript, the patient has been alive without recurrence or distant metastases for 6 months since the surgery. We mainly focus on the ultrasound imaging characteristics, especially its enhancement manifestations on CEUS. In this report, since this article is a case report, which is based on the clinical information of the patient and does not involve the patient's privacy, informed consent is not necessary. In addition, the patient agreed to publish the case. To the best of our knowledge, this report is the first to describe the CEUS patterns of the HCC combined with NEC. Herein, we report a case that provides novel insights that will improve clinicians' awareness of the clinical and ultrasound manifestations of this mixed tumor, resulting in improved diagnosis, treatment, and outcomes.

## Introduction

Two or more new tumor tissue structures may be detected simultaneously in several organs, which is called a mixed tumor. In the liver, most of the mixed carcinomas consist of a combination of hepatocellular carcinoma (HCC) and cholangiocarcinoma (1–3). However, the incidence of HCC with primary hepatic neuroendocrine carcinoma (NEC) is quite low. In fact, even the incidence of primary NEC is very uncommon, which is a rare variant of primary liver malignant tumor with unique clinicopathological characteristics (4). Therefore, the detection and understanding of the clinical manifestations and imaging findings of this rare mixed tumor are critically important for its timely diagnosis and treatment. In this paper, we present a case of HCC combined with NEC, with an emphasis on the ultrasound imaging and contrast-enhanced ultrasound (CEUS) findings.

## Case Presentation

**Figure 1:**
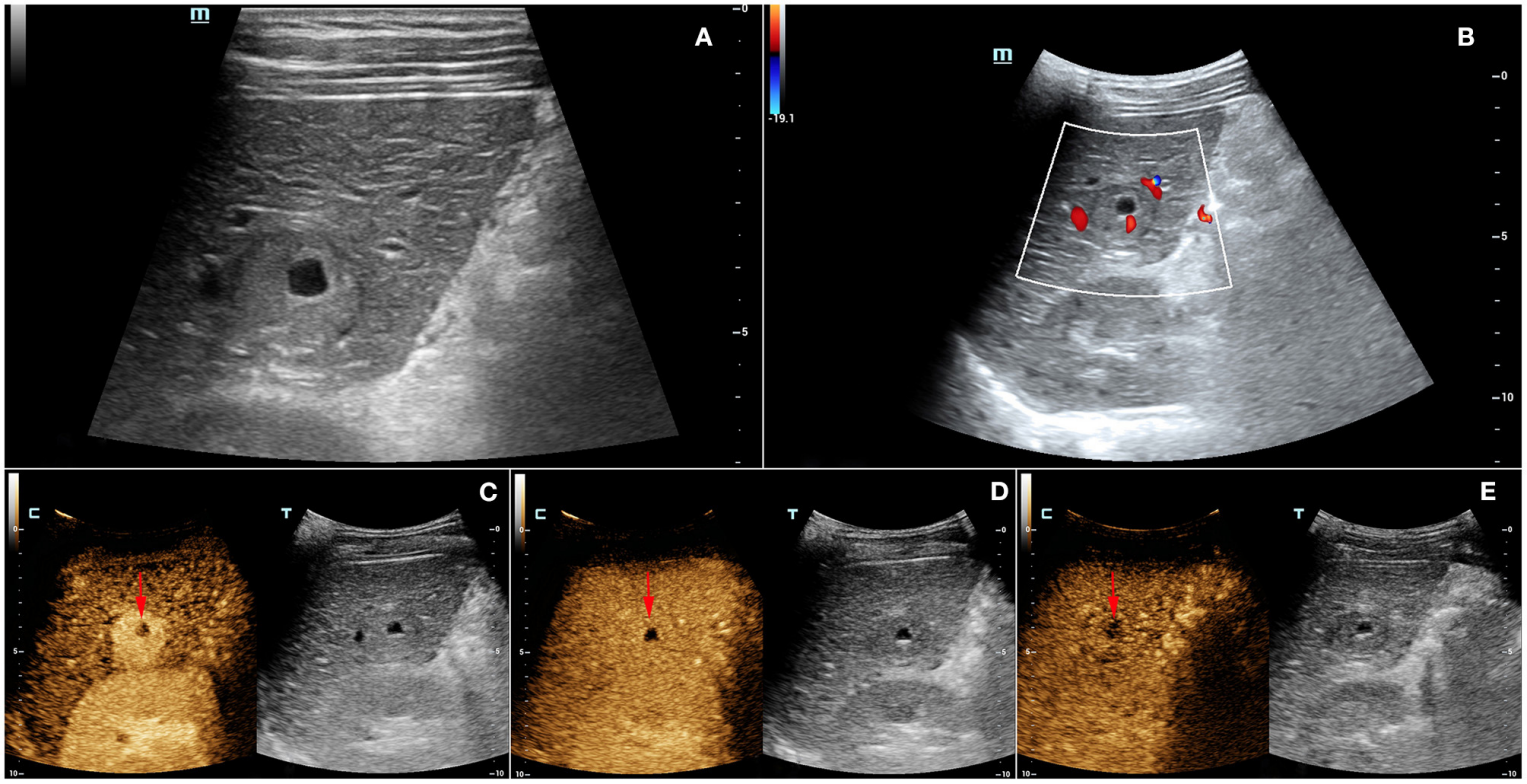
**(A)** Grayscale ultrasound showed a hypoechoic nodule with peripheral hypoechoic halo and centrally anechoic area in the hepatic segment VI. **(B)** Color Doppler flow imaging (CDFI) displayed dot-linear blood flow signals within the liver nodule. In the contrast-enhanced ultrasound (CEUS), **(C)** the solid component of the nodule presented rapidly homogeneous hyper-enhancement in the arterial phase. **(D)** The enhancement of the nodule was washed out slowly and gradually, with heterogenous mild enhancement in the portal phase. **(E)** In the late phase (4 min after contrast agent injection), the nodule showed slight hypo-enhancement, and the hypo-enhanced area was mainly located in the surrounding space. There was a small piece of non-enhanced area (arrows) within the lesion center in the whole three-phase imaging.

**Figure 2:**
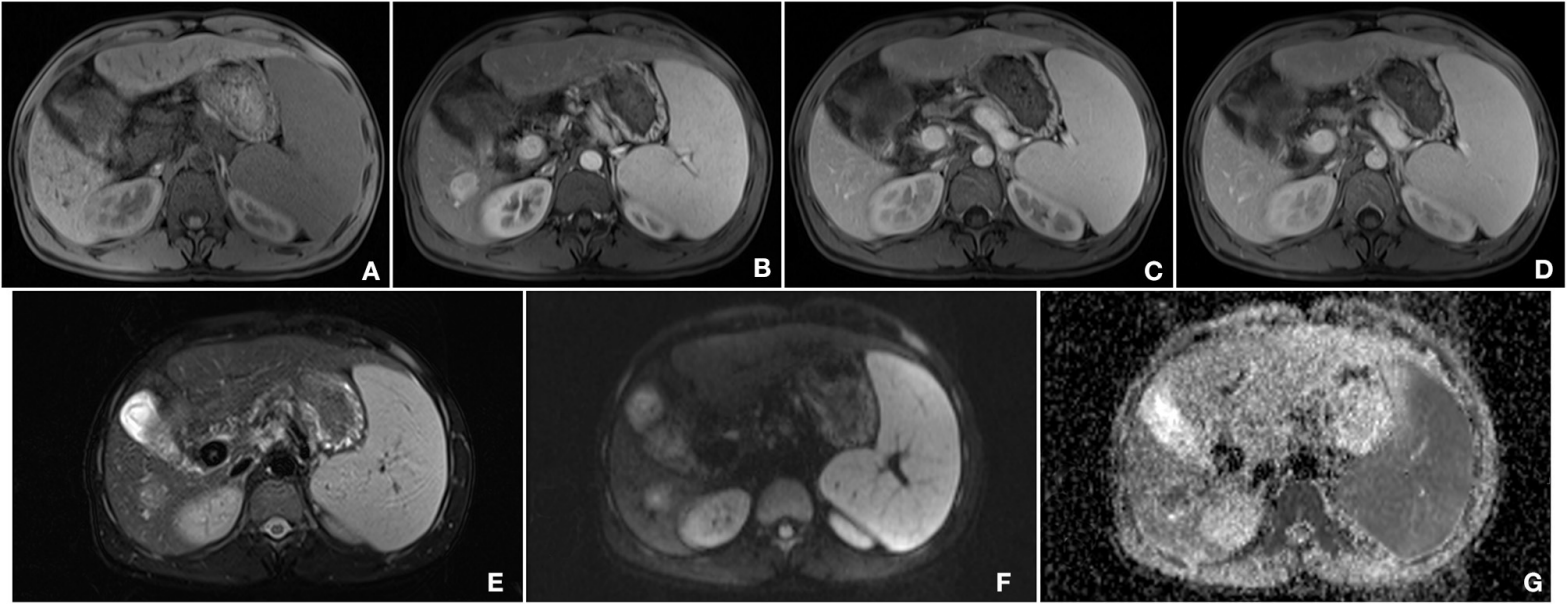
Findings of magnetic resonance imaging (MRI). **(A)** T1-weighted image of the nodule showed an iso-intensity lesion. **(B)** In the arterial phase, the lesion was significantly enhanced, and there was wash out both in the portal phase **(C)** and late phase **(D)**. **(E)** T2-weighted image of the tumor presented a slightly higher intensity. **(F)** Diffusion-weighted image of the tumor was a marked hyper-intensity. **(G)** ADC showed a low-signal lesion.

A 33-year-old male, who claimed to have had a 5-year history of liver cirrhosis with antiviral treatment and liver protection drugs, was admitted to our hospital due to a “liver space-occupying lesion” for the past 2 weeks. Physical examination revealed abdominal flatness and no tenderness point, but the spleen was swollen. Laboratory studies detected infection by hepatitis B virus and almost normal liver function. However, serum examinations detected a level of alpha-fetoprotein (AFP) of 403 ng/ml. Then, the patient underwent an abdominal ultrasound examination by a Resona7 ultrasound system (Mindray Medical International, Shenzhen, China) equipped with an SC6-1U (1–6 MHz) and L9-3 (3–9 MHz) transducer. The conventional B-mode ultrasound image showed a malformation of the liver; the capsule of the liver was not smooth, and the echo of the liver parenchyma was rough and heterogeneous. Meanwhile, a hypoechoic nodule with peripheral hypoechoic halo, located in the hepatic segment VI, was found. The lesion had an approximate size of 2.7 cm × 2.2 cm, with an almost regular shape and slightly clear margins; small patches of anechoic area were detected within the tumor (Figure 1A). Color Doppler flow imaging (CDFI) showed dot-linear blood flow signals within the liver nodule (Figure 1B). In view of the patient's high level of serum AFP and liver cirrhosis, the patient's doctor suggested undergoing CEUS for further diagnosis and obtained patient's consent. SC6-1U transducer was applied for CEUS. Dual-screen (on the screen are simultaneously displayed grayscale ultrasound and CEUS images) was used for real-time contrast-specific imaging at low mechanical index (the mechanical index setting was 0.078). The depth, gain, and focus were thoroughly adjusted for the optimal display according to the operator's habits. A 2.4-ml ultrasound contrast agent SonoVue (Bracco, Milan, Italy) suspension was injected through the left cubital vein followed by a flush with 5-ml saline. The timer was started when the contrast agent injection was completed. The target lesion and surrounding liver parenchyma were observed continuously for 5 min. According to well-established guidelines, arterial phase was defined as 10–30 s after contrast injection, the portal phase was 30–120 s, and the late phase was 121–360 s. All examinations were digitally recorded on the ultrasound system. In the arterial phase, the solid component of the nodule presented rapidly homogeneous hyper-enhancement (Figure 1C), without rim-like enhancement. Then, the enhancement of the nodule was washed out slowly and gradually. The nodule appeared heterogeneous, with mild enhancement in the portal phase (Figure 1D), and slight hypo-enhancement was detected in the late phase. The hypo-enhanced area was mainly located in the surrounding space (Figure 1E). Meanwhile, there was a small piece of non-enhanced area within the lesion center in the whole three-phase images. Therefore, the tumor was diagnosed as malignancy; combined with the history of liver cirrhosis, it was finally misdiagnosed as HCC. Additional dynamic liver magnetic resonance imaging (MRI) revealed a 2.6 cm × 2.2 cm-sized lobular contoured nodule in the liver parenchyma. The nodule showed high enhancement in the arterial phase and low density after wash-out of the contrast medium in the portal and delayed phases (Figure 2).

**Figure 3:**
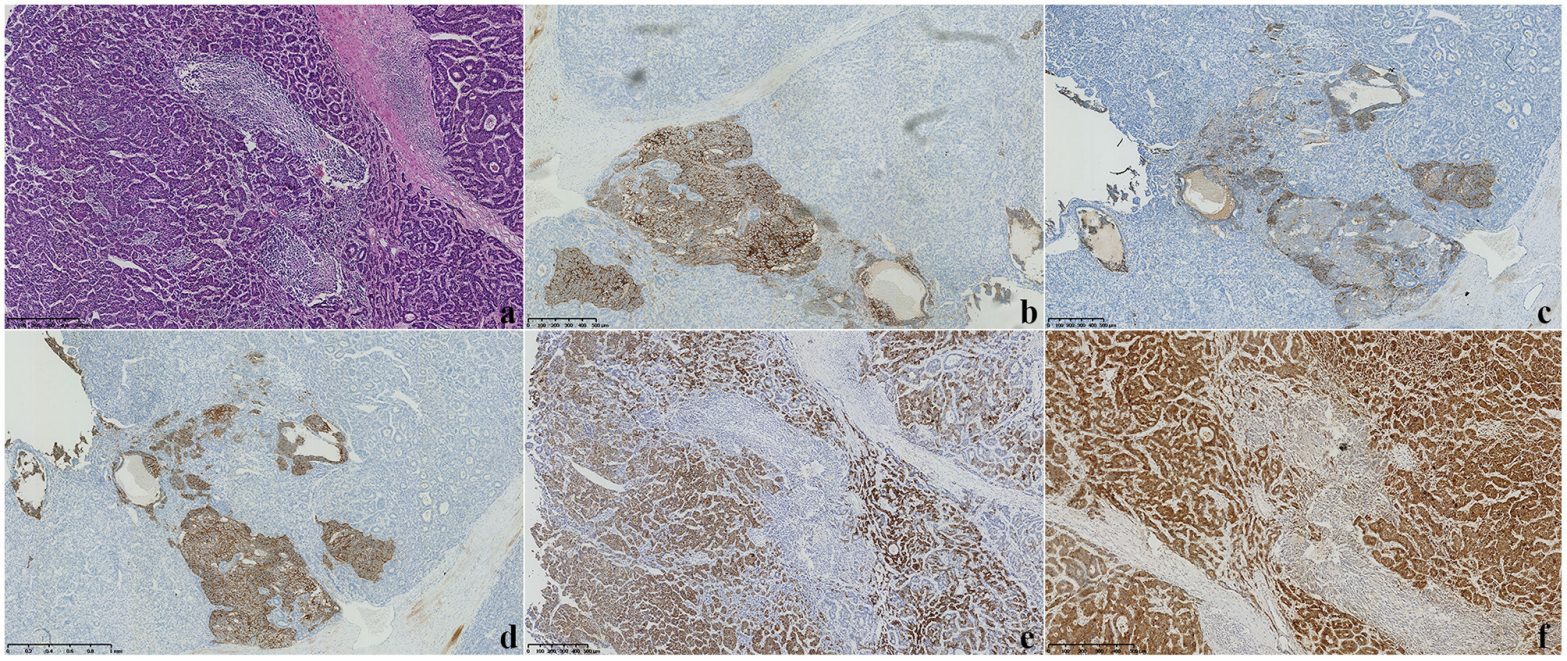
**(a)** Microscopy showed that the tumor consisted of two components: a hepatocellular carcinoma (HCC) and a neuroendocrine carcinoma (NEC) (H&E staining, ×100). **(b–f)** Immunohistochemical staining displayed CD56(+), CgA(+), Syn(+), HEPA(+), and GPC-3 expression (×100).

Soon afterward, middle liver resection, splenectomy, and cholecystectomy were performed. Severe liver cirrhosis changes were found during the operation. An intraoperative ultrasound revealed that the nodule was located in segment VI, extending to segment V, with an approximate size of 2.7 cm × 2.0 cm. No tumor changes were observed in the rest of the liver. Microscopic examination revealed that the tumor consisted of two components: a HCC and a NEC (Figure 3a). Immunohistochemistry showed positive CD56, positive chromogranin A (CgA), positive synaptophysin (Syn), strongly positive HEPA, and positive GPC-3 expression (Figures 3b–f). The non-neoplastic liver had stage-3 liver cirrhosis and portal fibrosis. Therefore, based on the combination of morphological and immunohistochemical analysis results, the patient was diagnosed with HCC combined with 2% NEC. Most of the tumor area was HCC, accounting for ~98% of the lesion. At the time of the preparation of this manuscript, the patient has been alive without recurrence or distant metastases for 6 months since the surgery, and he has had no other symptoms; his AFP level was normal at the 6-month follow-up examination.

## Discussion

The coexistence of HCC and NEC is rare as compared with the mixed type of HCC with cholangiocarcinoma in the liver, which is mainly due to the relatively low incidence of primary hepatic NEC. With the aim to investigate more comprehensively and understand better the clinical characteristics, imaging performance, and treatment, here, we present a case of a liver tumor with classical HCC combined with NEC. In this case, the patient was young, male, and had a history of hepatitis B, which is consistent with previous evidence (4–6). To sum up, the tumor of HCC combined with NEC was characterized by male patients with an underlying liver disease, such as chronic hepatitis or cirrhosis of unknown cause, sometimes fatty liver (7).

The precise diagnosis of this rare mixed tumor is based predominantly on postoperative pathological examination findings. The patient in the case was diagnosed with HCC of the liver complicated with NEC, established by postoperative pathology. The pathological diagnosis was based on the following findings: (1) morphologically, the tumor tissue was composed of two components, with HCC morphology and with NEC morphology. This tumor was a HCC with the features of a small NEC. The two types of tumor cells were separated by wide fibrous band, with no transition areas observed under a microscope; hence, a single disease could not explain the various morphologies; (2) immunohistochemical results: strongly positive HCC markers, HEPA(+) and GPC-3(+), and positive neuroendocrine markers, CgA(+), Syn(+), and CD56. (3) The tumor size was 2.5 cm ×2 cm, with most of the area occupied by the hepatocellular carcinoma tumor, accounting for ~98% of the whole tumor area. (4) The results of abdominal MRI did not support the diagnosis of metastasis to the liver from other sites. In addition, regarding the genetic analysis results of this mixed tumor, both tumor components contained genetic mutations in the CTNNB1 gene (S33F located in exon 3), which were shared in the PD-1, PGP, and SMO of this gene mutation (8).

The preoperative diagnosis of a mixed tumor is a major challenge. Our literature review showed that combined HCC and NEC, on the one hand, are similar to HCC in their clinical and hematological manifestations, such as the possible combination of hepatitis B or C, cirrhosis, and elevated levels of AFP in the blood. On the other hand, the vascular and extrahepatic metastases of the mixed tumor are similar to those of other mixed tumors such as cholangiocarcinoma, and it is difficult to obtain an accurate preoperative diagnosis. In terms of imaging findings, there have been few reports on ultrasound and CEUS. In this case, we report CEUS findings of a mixed tumor for the first time. Our analysis revealed that mixed carcinoma needs to be differentiated from HCC. These features of CEUS in the present case were similar to those of HCC, and the patient had a history of hepatitis B and cirrhosis in the liver. Thus, the mixed HCC–NEC diagnosis is difficult to make or differentiate from that of HCC alone. Retrospective analysis results showed that in this case, conventional ultrasound and CEUS revealed patches of cystic changes within the tumor and relatively regular morphology of the area. However, in small HCC ( ≤ 3 cm), similar cystic changes within the tumor are rarely found (9–12). Therefore, this feature may serve as a point of differentiation between mixed carcinoma and HCC. Certainly, more data are needed for further verification. In addition, combined with the CEUS findings in this case, the mixed HCC–NEC also needs to be differentiated from other non-HCC nodules with CEUS manifestations (fast-forward and fast-out) that may be similar to those of HCC, such as liver tuberculosis and liver carcinoid (13, 14). Differential diagnosis with primary NEC and metastatic NEC is required (15). It is important to note that the liver is the most common site of metastasis of NEC. Other possible organs also need to be examined when NEC metastases to the liver are suspected. In this case, no neoplastic lesions other than the liver were found.

The prognosis and treatment of HCC accompanied by NEC are unclear due to the limited number of cases reported. Our analysis of the literature found that the presence of NEC components implicates the possibility of aggressive behavior of the mixed-type tumor (5, 16, 17). Considering the different prognosis of patients in different case reports, it is speculated that the NEC component may be responsible for the outcomes in these cases (4, 18, 19). The treatment is also comprehensive based on hepatectomy, combined with multiple treatment modes, such as radical resection, lymph node dissection, and adjuvant treatment, aimed at the provision of the most favorable outcomes, including long-term survival. Other therapies can be considered in case of tumor recurrence, such as transcatheter arterial chemoembolization (TACE) and percutaneous ethanol injection therapy (PEIT) (10, 20).

In summary, although a mixed HCC and NEC tumor is a rare condition, in patients with chronic liver disease, the rare possibility of HCC–NEC needs to be considered in the differential diagnosis, regardless of the presence of cirrhosis even if diagnostic clinical signs are not manifested and imaging features are lacking. Although the final diagnosis is based mainly on pathological and immunohistochemical examinations, ultrasonography, including CEUS, is widely used for detection of liver lesions.

Our case is important as it is the first to highlight the value and utility of CEUS for diagnosis in patients with HCC tumor combined with NEC. Our report provides novel insights that can serve as a basis for further research and will improve clinicians' awareness of the clinical and ultrasound manifestations of this mixed tumor, resulting in improved diagnosis, treatment, and outcomes.

## Data Availability Statement

The raw data supporting the conclusions of this article will be made available by the authors, without undue reservation.

## Ethics Statement

Written informed consent was obtained from the individual(s) for the publication of any potentially identifiable images or data included in this article.

## Author Contributions

HW collected, conducted, and interpreted the patient data and contributed significantly to the writing of the initial manuscript. WL revised the paper and had primary responsibility for the final content. ZW performed and analyzed pathological data. YL contributed to the conception of the study. DY was the major contributor in coordinating patient care and helped perform the analysis with constructive discussions. The final manuscript was read and approved by all authors.

## Conflict of Interest

The authors declare that the research was conducted in the absence of any commercial or financial relationships that could be construed as a potential conflict of interest.

## Publisher's Note

All claims expressed in this article are solely those of the authors and do not necessarily represent those of their affiliated organizations, or those of the publisher, the editors and the reviewers. Any product that may be evaluated in this article, or claim that may be made by its manufacturer, is not guaranteed or endorsed by the publisher.

## Abbreviations

AFP: alpha-fetoprotein
HCC: hepatocellular carcinoma
NEC: neuroendocrine carcinoma
CEUS: contrast-enhanced ultrasound
TACE: transcatheter arterial chemoembolization
PEIT: percutaneous ethanol injection therapy.
